# APOL1 risk variants cause podocytes injury through enhancing endoplasmic reticulum stress

**DOI:** 10.1042/BSR20171713

**Published:** 2018-08-29

**Authors:** Hongxiu Wen, Vinod Kumar, Xiqian Lan, Seyedeh Shadafarin Marashi Shoshtari, Judith M. Eng, Xiaogang Zhou, Fang Wang, Haichao Wang, Karl Skorecki, Guolan Xing, Guisheng Wu, Huairong Luo, Ashwani Malhotra, Pravin C. Singhal

**Affiliations:** 1 Key Laboratory for Aging and Regenerative Medicine, School of Pharmacy, Southwest Medical University, Luzhou, Sichuan, China; 2 Feinstein Institute for Medical Research and Hofstra Northwell Medical School, Manhasset, NY, U.S.A.; 3 Department of Emergency Medicine, North Shore University Hospital, Manhasset, NY, U.S.A.; 4 Nephrology and Molecular Medicine, Technion Institute of Technology and Rambam Medical Center, Haifa, Israel; 5 Department of Nephrology, First Affiliated Hospital of Zhengzhou University, Zhengzhou University Institute of Nephrology, Henan, China

**Keywords:** APOL1, ER stress, Podocyte

## Abstract

Two coding sequence variants (G1 and G2) of Apolipoprotein L1 (*APOL1*) gene have been implicated as a higher risk factor for chronic kidney diseases (CKD) in African Americans when compared with European Americans. Previous studies have suggested that the APOL1 G1 and G2 variant proteins are more toxic to kidney cells than the wild-type APOL1 G0, but the underlying mechanisms are poorly understood. To determine whether endoplasmic reticulum (ER) stress contributes to podocyte toxicity, we generated human podocytes (HPs) that stably overexpressed APOL1 G0, G1, or G2 (Vec/HPs, G0/HPs, G1/HPs, and G2/HPs). Propidium iodide staining showed that HP overexpressing the APOL1 G1 or G2 variant exhibited a higher rate of necrosis when compared with those overexpressing the wild-type G0 counterpart. Consistently, the expression levels of nephrin and podocin proteins were significantly decreased in the G1- or G2-overexpressing cells despite the maintenance of their mRNA expressions levels. In contrast, the expression of the 78-kDa glucose-regulated protein ((GRP78), also known as the binding Ig protein, BiP) and the phosphorylation of the eukaryotic translation initiation factor 1 (eIF1) were significantly elevated in the G1/HPs and G2/HPs, suggesting a possible occurrence of ER stress in these cells. Furthermore, ER stress inhibitors not only restored nephrin protein expression, but also provided protection against necrosis in G1/HPs and G2/HPs, suggesting that APOL1 risk variants cause podocyte injury partly through enhancing ER stress.

## Introduction

Clinical reports have demonstrated that African Americans develop higher rates of progressive nephropathy including focal segmental glomerulosclerosis (FSGS), hypertension-attributed chronic kidney disease (CKD), and HIV-associated nephropathy (HIVAN) when compared with European Americans [1,2]. This major health disparity is strongly associated with two derived coding sequence variants (G1 and G2) in the Apolipoprotein L1 (*APOL1*) gene (with an ancestral non-risk designated as G0) [3–5]. To evaluate the underlying mechanisms, we and others have previously demonstrated that APOL1 G1 and G2 are more cytotoxic in both podocytes and HEK293T cells when compared with the wild-type G0 [6–10]. We observed that the G1 and G2 could increase the lysosomal membrane permeability, causing podocyte necrosis [6]; Ma et al. [9] reported that these two variants impaired mitochondrial function. Moreover, Beckerman et al. [11] showed that APOL1 risk alleles transgenic mice developed renal lesions similar to human APOL1 nephropathies; they also reported that the podocytes from the risk allele transgenic mice had impaired endolysosmal trafficking and autophagic flux, and inflammasome activation [11]. However, the underlying molecular mechanisms for APOL1-associated nephropathy are still poorly understood.

Podocytes are terminally differentiated and highly specialized epithelial cells in the Bowman’s capsule of the kidneys. They wrap around capillaries of the glomerulus, and extend foot processes to form a filtration barrier. Podocytes play a crucial role in the regulation of glomerular filtration function in both under physiological and pathological states; the majority of the proteinuric diseases, including focal glomerular sclerosis and HIVAN, are associated with podocyte injury [12–15]. Localization studies have shown that APOL1 is expressed in podocytes [16], suggesting that podocytes might be a target of APOL1 risk variants. Studies from our group as well as others have demonstrated that APOL1 risk variants cause a greater injury to podocytes when compared with the non-risk APOL1 [6,11,17,18].

Endoplasmic reticulum (ER) is an organelle with highly folded tubular membranes within the cytoplasm of the eukaryotic cell, and it is the site of cellular chemical reactions and involved in the transport of materials. It is known as smooth ER or rough ER, if ribosomes are attached. Rough ER is the initial point of the biosynthetic pathways of phospholipids, carbohydrate chains, and proteins, while smooth ER synthesizes steroids and regulates calcium concentration. Newly synthesized polypeptides translocate into the lumen of the ER and attain a 3D conformation after undergoing protein folding and post-translational modifications (for example, disulphide bond formation and N-linked glycosylation). Proper protein processing requires a carefully orchestrated series of events, and improper protein folding can lead to the build-up of misfolded proteins. The accumulation of misfolded/unfolded proteins in the ER and alterations in calcium homeostasis lead to ER stress, which leads to the unfolded protein response (UPR, a series of integrative stress pathways). ER stress has been widely demonstrated to contribute to both glomerular and tubular cell injuries in kidney diseases [19–21]; moreover, it has been shown to be a mediator of podocyte apoptosis [22–25].

Since APOL1 G0, G1, and G2 proteins show difference in only a few amino acid residues, we speculate the risk variants G1 and G2 proteins form different configurations from G0 due to the mutations. G1 and G2 may be considered as ‘misfolded proteins’, which carry potential to stimulate ER stress, contributing to cell injury. Whether APOL1 risk variants cause increased ER stress in podocytes has not been studied so far. In the present study, we determined the role of ER stress in the induction of APOL1 variants induced podocyte injury.

## Materials and methods

### Reagents

Salubrinal (Cat# SML0951), 4-phenylbutyrate (4-PBA, Cat# SML0309), and cisplatin (Cat# C2210000) were purchased from Sigma–Aldrich (St. Louis, MO).

### Generation and culture of APOL1 stable podocyte cell lines

To establish human podocyte (HP) cell lines stably expressing APOL1, we cloned the cDNAs of APOL1 variants G0, G1, and G2 into the retroviral vector pBABE-eGFP at BamHI and EcoRI sites, and then prepared the retrovirus by using these recombinant vectors, following the methods described in a previous report [26]. HP cell line, which was generated by Saleem et al. [27], was transfected with the prepared retrovirus. The cells were highly diluted and were planted on to 96-well plates to generate cell colonies. Positively transfected cell colonies were confirmed by GFP visualization under fluorescence microscope, and were then isolated, lifted, and used for the determination of APOL1 expression through real-time PCR and Western blotting. Cell colonies stably expressing APOL1 G0, G1, and G2 were expanded and designated as APOL1-G0/HPs, G1/HPs, and G2/HPs. Empty vector pBABE-eGFP was also transduced into HPs to generate the control cell line APOL1-Vec/HPs. These cells were cultured as previously reported [6,27,28]. Briefly, podocytes proliferated in the growth medium containing RPMI 1640 supplemented with 10% FBS, 1× penicillin-streptomycin, 1 mM l-glutamine, and 1× insulin, transferrin, and selenium (ITS) (Invitrogen, Grand Island, NY) at permissive temperature (33°C). When the cells reached approximately 80% confluence, they were transferred to 37°C for differentiation in a medium without ITS for 7 days.

### RT-PCR

Total RNA was isolated from HPs using TRIzol reagent (Invitrogen). Five micrograms of total RNA were reverse transcribed using the first-strand synthesis system (Invitrogen). PCR was performed by using Platinum PCR SuperMix High Fidelity (Invitrogen). Sequences of primers for human *nephrin* and *podocin* were GTCTGCACTGTCGATGCCAATC (Nephrin-FW), CACCCTGGTGGTATGTGTGCTC (Nephrin-RV), AATCCAAGGCAACCTTTGCATC (Podocin-FW), and CCTTTGGCTCTTCCAGGAAGCA (Podocin-RV). GAPDH was used as internal control, and its forward primer was TCAACGGATTTGGTCGTATTGG, and reverse primer was AGTCTTCTGGGTGGCAGTGATG. Amplification was performed at 95°C for 5 min, followed by 30 cycles at 94°C for 1 min, 55°C for 30 s, 68°C for 30 s with a final extension cycle for 5 min at 68°C. DNA samples were visualized by ChemiDo Imaging Systems (Bio-Rad) after electrophoresis with 2% agarose gel.

Real time-PCR was performed in a Prism 7900HT sequence-detection system (Applied Biosystems, Foster City, CA, U.S.A.). Relative mRNA levels were determined and standardized with a GAPDH internal control using comparative ΔΔ*C*_T_ method. Sequences of primers for human *APOL1* were ATCTCAGCTGAAAGCGGTGAAC (APOL1-FW), and TGACTTTGCCCCCTCATGTAAG (APOL1-RV). The primers for GAPDH were the same as mentioned above.

### Nucleotide sequencing

To determine the APOL1 variant type of HP cell line, we extracted the genomic DNA from undifferentiated podocytes with DNeasy Blood and Tissue Kit (Qiagen, Germantown, U.S.A., Cat# 69506), and used it as the template for nucleotide sequencing at DNA Sequencing Facility, Albert Einstein College of Medicine, New York, U.S.A.. The primer for sequencing was APOL1-FW as mentioned above.

To determine the nucleotide sequences of the mRNAs from APOL1-G0/HPs, G1/HPs, and G2/HPs, we extracted total RNA from differentiated cells, and prepared cDNAs as mentioned above. We then used these cDNAs as templates to amplify the DNA fragments through PCR. The primers were APOL1-FW and APOL1-RV (as mentioned above). The PCR products were purified with QIAquick PCR Purification Kit (Qiagen, Germantown, U.S.A., Cat# 28104), and then were used as template for DNA sequencing.

### Western blot analysis

Western blotting was performed using established methodology [6,28]. Briefly, cells were washed with PBS and lyzed in RIPA buffer. Proteins (20–30 μg) were separated by SDS/PAGE (12% gel) and then transferred on to an immunoblot PVDF membrane (Bio-Rad, Hercules, CA). After blocking in PBS/Tween (0.1%) with 5% non-fat milk, the membrane was incubated with primary antibodies overnight at 4°C followed by horseradish peroxidase-conjugated secondary antibodies (Santa Cruz, 1:3000) and then developed using ECL solution (Pierce). Primary antibodies used were mouse anti-APOL1 (Proteintech, 66124-1-lg, 1:1000), rabbit anti-nephrin (Abcam, ab58968, 1:1000), rabbit anti-podocin (Sigma, P0372, 1:2000), rabbit anti-GRP78 binding Ig protein (BiP) (Abcam, ab21685, 1:1000), rabbit anti-p-eIF2α (Cell Signaling Technology, #9721, 1:1000), and mouse anti-actin (Santa Cruz Biotechnology, sc-8432, 1:3000). For protein expression quantitation, the films were scanned with a CanonScan 9950F scanner and the acquired images were then analyzed using the public domain NIH image program (http://rsb.info.nih.gov/nih-image/).

### Propidium iodide and Hoechst staining

The Propidium iodide (PI) and Hoechst staining was performed as reported earlier [6]. Briefly, after appropriate treatment, the culture medium was removed from the cells, and fresh medium containing Hoechst 33342 (10 μg/ml) was added. Addition of cisplatin (40 μM) to the medium for 48 h was used as a positive control for necrosis. Cells were subsequently incubated for 10 min at 37°C. Then, PI solution was added and the cell dishes were put on ice for 7 min. Cell images were obtained immediately with a ZEISS microscope (Carl Zeiss MicoImaging GmbH, Jena, Germany) equipped with a digital imaging system. Fifteen fields were randomly selected, and the images were obtained by a blinded investigator.

### Immunofluorescent microscopy

HPs were differentiated at 37°C for 7 days, and were rinsed with PBS and fixed with 4% paraformaldehyde for 15 min. Then the cells were blocked with blocking buffer containing 2% BSA and 0.1% Triton X-100 in PBS for 1 h at room temperature. Samples were incubated overnight with rabbit anti-GRP78 BiP (Abcam, ab21685, 1:200) and mouse anti-APOL1 (Proteintech, 66124-1-lg, 1:200) in blocking buffer at 4°C, followed by washing with 0.1% Triton X-100 in PBS for three times. Subsequently, samples were incubated for 60 min at room temperature with Alexa Fluor 488 goat anti-rabbit IgG and Alexa Fluor 594 goat anti-mouse IgG. To label nuclei, cells were then counterstained with Hoechst 33342 (Sigma–Aldrich). Morphological changes were visualized and captured with a ZEISS microscope (Carl Zeiss MicoImaging GmbH, Jena, Germany) equipped with a digital imaging system.

### Statistical analyses

Data were presented as means ± S.D. unless otherwise noted. All experiments were repeated at least three times with duplicate or triplicate samples in each assay. All data were evaluated statistically by the ANOVA, followed by Newman–Keuls multiple comparison tests using software (Prism 4.0, GraphPad Software). In the case of single mean comparison, data were analyzed by *t* test. *P*-values <0.05 were regarded as statistically significant.

## Results and discussion

### Generation and characterization of APOL1 stable cell lines

**Figure 1:**
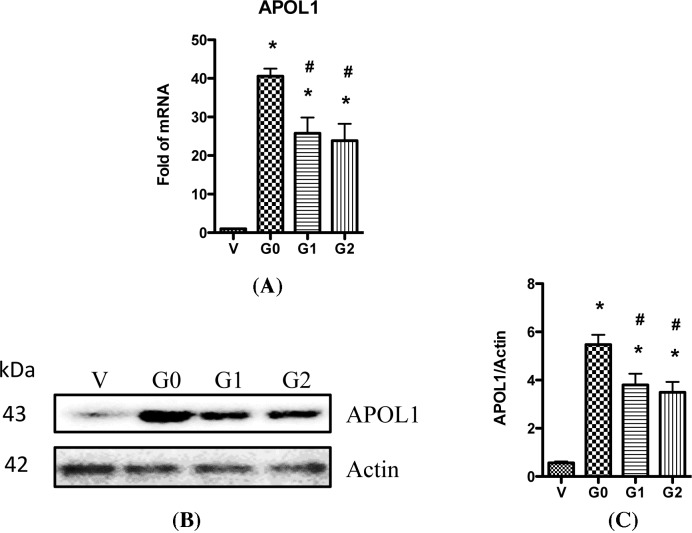
APOL1 expression in HP cell lines (**A**) Total RNAs were prepared from differentiated HPs (APOL1-Vec/HPs, G0/HPs, G1/HPs, and G2/HPs), and were used for real-time PCR to detect the expression of *APOL1* mRNA. GAPDH was used as loading control. (**B**) Cell lysates were collected from differentiated HPs (APOL1-Vec/HPs, G0/HPs, G1/HPs, and G2/HPs), and subjected to Western blotting to detect the expression of APOL1 protein. Representative files were selected to show the protein expression. (**C**) Densitometry analysis with ImageJ software was performed on scanned Western blotting films, and band intensities were calculated and plotted as a ratio of antibody intensity normalized to actin. Values are means ± S.D. represent three independent samples. *P<0.05 compared with APOL1-Vec/HPs (V), and ^#^*P*<0.05 compared with APOL1-G0/HPs (G0).

First, we characterized the APOL1 variant type in a podocyte cell line generated by Saleem et al. by genomic sequencing of the *APOL1* gene [27]. Results showed that the sequence was exactly the same as of APOL1 G0 described in GenBank at NM_145343 (data not shown), confirming that it was G0 in this podocyte cell line. We then used it to generate HP cell lines that overexpressed APOL1-G0, G1, or G2, respectively. To confirm the elevated expression of various APOL1 variants in the podocytes, we collected RNAs from these cells and performed real-time PCR. Results showed that *APOL1* mRNA were increased by 41-, 26-, and 23-folds in APOL1-G0/HPs, G1/HPs, and G2/HPs, respectively, when compared with APOL1-Vec/HPs (Figure 1A). We also confirmed the protein expression of APOL1 in these cells by Western blotting after collecting the cell lysates; APOL1 protein expression were significantly increased in APOL1-G0/HPs, G1/HPs, and G2/HPs when compared with vector control (Figure 1B,C).

**Figure 2:**
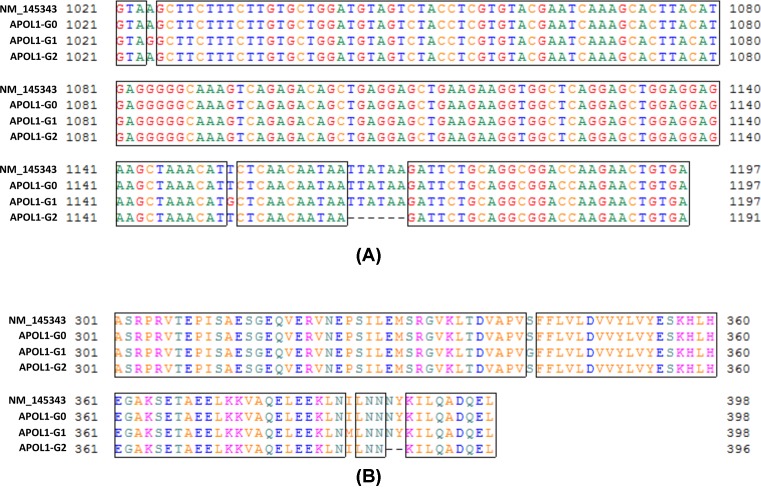
Determination of APOL1 variant types of HP cell lines Total RNAs from APOL1-G0/HPs, G1/HPs, and G2/HPs were extracted, and then were used to prepare cDNAs. PCR was performed to amplify the *APOL1* gene, and the products were used for the determination of the nucleotide sequences. The sequence of APOL1-G0 from GenBank (ID number: NM_145343) was used as control to analyze the alignments of nucleotide sequence (**A**) and speculated amino acid sequence (**B**).

To validate, these cell lines were genuinely expressing APOL1-G0, G1, and G2, respectively, we collected their total RNAs, prepared cDNAs, and then determined the nucleotide sequences of their APOL1 variants. Results showed that in APOL1-G0/HPs, the sequence was completely the same as that in GenBank at NM_145343; while in APOL1-G1/HPs, the adenine1 (A) at +1024 and the uracil (U) at +1052 of the sequence were replaced with guanine (G), and in APOL1-G2/HPs, the six bases of UUAUAA (+1064 to +1069) were missed (Figure 2A). Correspondingly, in the speculated amino acid sequences, the serine at 342 (S342) and isoleucine at 384 (I384) were replaced with Gly^342^ (G342) and Met^384^ (M384) in G1; while the N388Y389 were missed in G2 (Figure 2B). These results perfectly matched the description of APOL1 G1 and G2 variants [29,30].

Since the expression of *APOL1* mRNAs from APOL1-G0/HPs, G1/HPs, and G2/HPs were much higher than that from APOL1-Vec/HPs (Figure 1A), we believe that in APOL1-G1/HPs and G2/HPs, the dominant *APOL1* mRNAs were from their newly inserted APOL1 expression cassettes, but not from the constitutive *APOL1* gene. Similarly, the dominant APOL1 proteins from these APOL1-G1/HPs and G2/HPs were APOL1-G1 and G2, respectively, and the constitutive APOL1-G0 protein contents in these two cell lines were negligible.

To examine whether the cell line could express APOL1 stably, we passaged these four cell lines for over ten generations, and then detected the expression of *APOL1* mRNA and protein. Results showed that the expression of APOL1 in all these cell lines didn’t significantly chang after so many passages (data not shown), indicating that the expression of these APOL1 variants was stable.

Interestingly, we found that both the mRNA and protein of APOL1-G0 were relatively higher than APOL1-G1 and G2. This might be due to the stronger toxicity of APOL1-G1 and G2 than G0, which led to the partial inhibition of the transcription and translation through feedback.

### APOL1 risk variants increase podocyte injury

**Figure 3:**
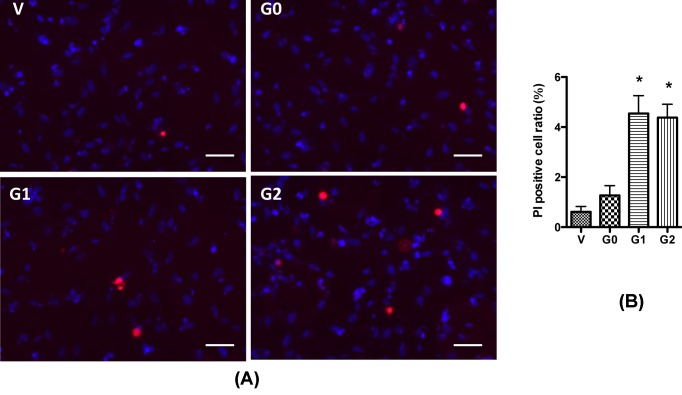
APOL1 risk variants cause podocyte injury HPs (APOL1-Vec/HPs, G0/HPs, G1/HPs, and G2/HPs) were differentiated for 7 days, and then were subjected to PI/Hoechst staining. Cell images were obtained with a microscope by a blinded investigator. Representative pictures (**A**) were selected to show the necrotic (red) cells, and the ratio of these cells were calculated (**B**) from randomly selected 15 scopes. *P<0.05 compared with APOL1-G0 HP. Scale bar: 50 µm.

Then we examined whether the expression of various APOL1 variants would divergently cause injury to podocytes. Since APOL1 variants have been demonstrated to cause cell swelling in podocytes and HEK293T cells [6–8], we examined the number of swollen cells in stably APOL1-expressing cells under a light microscope. Although in our previous studies, we observed a large number of swollen cells in podocyte transduced with APOL1-expressing lentivirus [6], any increase in the number of swollen podoctyes was not observed in stably APOL1-expressing cell lines. This might be due to the relatively lower expression of *APOL1* mRNA and protein when compared with our previous studies with lentivirus system. Consistent with our observation, O’Toole et al. [31] also reported that APOL1 risk variants caused cell toxicity to HEK293 cells only when expressed at higher levels. To determine whether APOL1 risk variants cause injury to the podocytes, we performed PI staining to count the necrotic cells. We found that G1 and G2 expressing cells showed a significant increase in the number of necrotic cells when compared with vector and G0 (Figure 3). As expected, G0 hardly showed any increase in necrotic cells when compared with vector controls.

**Figure 4:**
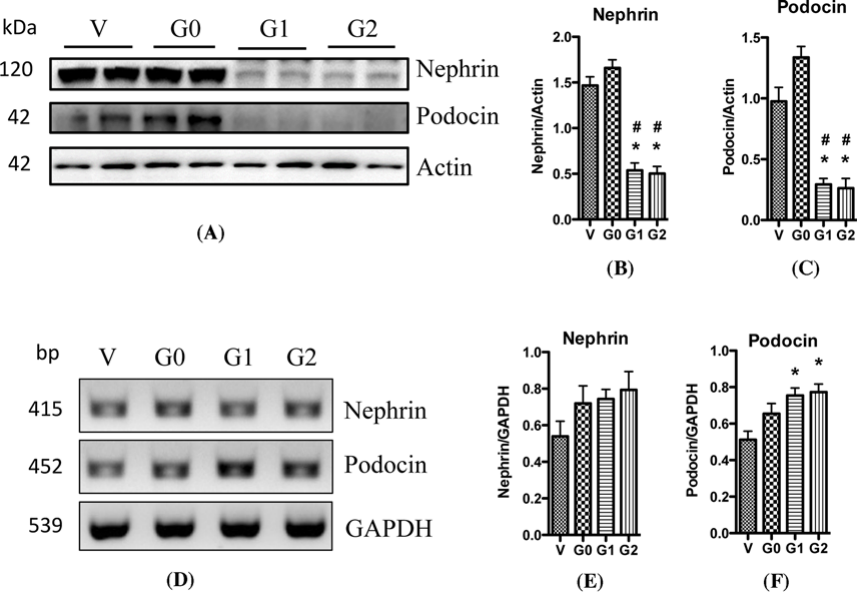
APOL1 risk variants affect the expression of nephrin HPs (APOL1-Vec/HPs, G0/HPs, G1/HPs, and G2/HPs) were differentiated for 7 days, and the cell lysates were collected and were subjected to Western blotting to detect the expression of nephrin and podocin. The films were scanned with a scanner, and representative gels were selected to show the protein expression (**A**). The acquired images were then analyzed using the public domain NIH image program, and the quantitation results (mean ± S.D.) of nephrin (**B**) and podocin (**C**) represent three independent samples. Actin was used for loading control. *P<0.05 compared with control (APOL1-Vec HP), and ^#^*P*<0.05 compared with APOL1-G0 HP. Total RNAs were prepared from differentiated HPs (APOL1-Vec/HPs, G0/HPs, G1/HPs, and G2/HPs), and were used for RT-PCR to detect the mRNA expression of nephrin and podocin. GAPDH was used as loading control. After electrophoresis with 2% agarose gel, DNA bands were visualized by ChemiDo Imaging Systems. Representative images were selected to display the DNA bands (**D**), and the quantitation results (mean ± S.D.) of nephrin (**E**) and podocin (**F**) represent three independent samples. *P<0.05 compared with control (APOL1-Vec/HP), and ^#^*P*<0.05 compared with APOL1-G0/HP.

Nephrin plays a key role in the maintenance of slit diaphragm (SD) structure. It comes into contact with many other SD proteins and triggers important cell signaling pathways in podocytes. A decrease in nephrin expression in podocytes is associated with kidney diseases including diabetic nephropathy and HIVAN [32,33]. Moreover, animal models with nephrin knockout/knockdown are associated with massive proteinuria [34,35]. Besides nephrin, podocin is another SD protein that serves as a scaffolding protein. Mutations in the podocin gene *NPHS2* can cause nephrotic syndrome characterized by renal lesions in the form of FSGS or minimal change disease (MCD) [36]. To evaluate the role of APOL1 variants in the modulation of expression levels of nephrin and podocin, we evaluated the expression of these proteins in podocytes overexpressing APOL1-G0 and APOL1 variants G1 and G2 by Western blotting analysis. As shown in Figure 4, overexpression of APOL1 with wild-type G0 did not change nephrin and podocin protein expression when compared with vector control; however, APOL1 G1 and G2 decreased their protein expressions (Figure 4A–C). These results further confirmed our hypothesis that APOL1 risk variants cause kidney cell injury through gain of toxicity.

Then we asked whether APOL1 risk variants also affect the transcription levels of nephrin and podocin. RT-PCR studies revealed that APOL1 G0 did not alter the mRNA levels of either of nephrin or podocin. On the other hand, APOL1 G1 and G2 displayed a tendency to increase mRNA levels of these genes (Figure 4D–F). Furthermore, we performed real-time PCR to compare the mRNA levels; as expected, there was no difference in nephrin and podocin expression amongst different groups (data not shown). Taken together, these results demonstrate that APOL1 risk variants cause podocyte injury, and they impair nephrin and podocin expression predominantly at the translational level.

### APOL1 risk variants increase ER stress in podocytes

Glucose-regulated protein 78 (GRP78), also known as BiP, is an ER specific molecular chaperone. It binds to newly synthesized proteins as they are translocated into the ER, and maintains them in a competent state for subsequent folding and oligomerization. Since GRP78 is a key regulator of the UPR, it is often regarded as an ER stress marker. Increasing evidence suggest that ER stress contribute to glomerular and tubular cell injury in kidney diseases [19–21]. In response to ER stress, three UPR signal transduction pathways are initiated by three transmembrane proteins: protein kinase-like ER kinase (PERK), activating transcription factor 6 (ATF6), and inositol requiring 1 (IRE1) [22]. The subsequence of activation of PERK is to cause the phosphorylation of eukaryotic translation initiation factor (eIF)-2α, which further causes the down-regulation of translation. The relationship between APOL1 risk variants and ER stress in podocyte has not been determined yet.

**Figure 5:**
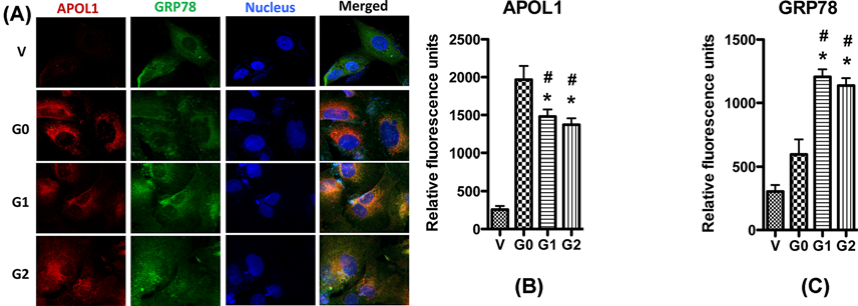
Immunocytochemistry of APOL1 and GRP78 in podocyte HPs (APOL1-Vec/HPs, G0/HPs, G1/HPs, and G2/HPs) were differentiated for 7 days, and then were immunolabeled with antibodies to APOL1 (red) and GRP78 (green). Nuclei were stained with Hoechst 33342 (blue). Representative pictures were selected to show the images (**A**), and the quantitation results (mean ± S.D.) of APOL1 (**B**) and GRP78 (**C**) represent three independent samples. *P<0.05 compared with control (APOL1-Vec HP), and ^#^*P*<0.05 compared with APOL1-G0 HP.

In the present study, we evaluated the effect of APOL1 variants on the ER stress. First, we performed immunocytochemistry to detect the distribution of APOL1 proteins in HPs. GRP78 is a chaperone which is localized in the ER, and is regarded as a biomarker for ER. As shown in Figure 5, APOL1 is co-localized with GRP78 in all cell lines, indicating that APOL1 locates in ER. We also found that when compared with vector, APOL1G1/G2, but not APOL1G0, significantly increased the expression of GRP78.

**Figure 6:**
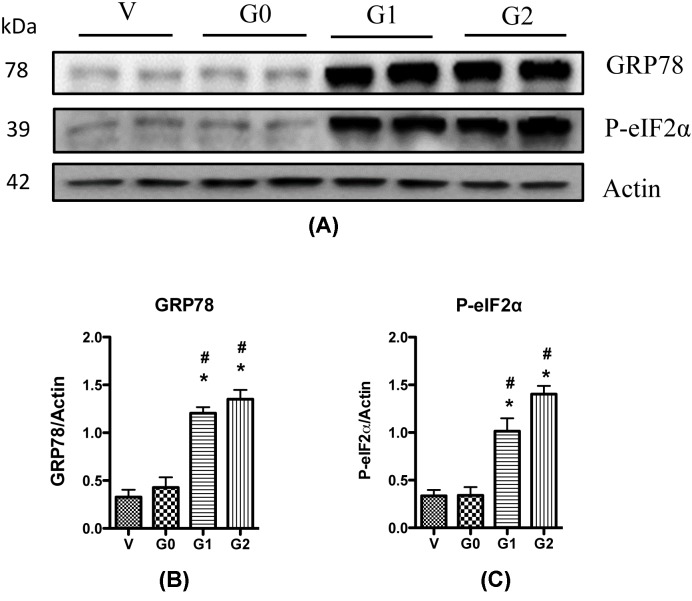
APOL1 risk variants enhance ER stress Cell lysates were collected from differentiated HPs (APOL1-Vec/HPs, G0/HPs, G1/HPs, and G2/HPs), and were subjected to Western blotting to detect the expression of GRP78 and p-eIF-2α. β-actin was used as an internal loading control. Representative gels are shown in (**A**). The protein bands were scanned and the acquired images were analyzed using the public domain NIH image program for data quantitation. Expression of GRP78 (**B**) and p-eIF-2α (**C**) were normalized to β-actin. Data are presented as fold of control expression. *P<0.05 in comparison with control (vector), and ^#^*P*<0.05 in comparison with APOL1-G0.

To confirm these observations, we performed Western blotting to determine the changes of GRP78 expression and the phosphorylation of eIF-2α, another ER stress biomarker, in the four podocyte cell lines. Results showed that the APOL1G0 did not affect GRP78 expression, but G1 and G2 increased its expression (Figure 6); similarly, the phosphorylation of eukaryotic translation initiation factor 1 (eIF1) was significantly elevated in the G1/HPs and G2/HPs, but not in the G0/HPs (Figure 6). Combined together, these results suggest the G1 and G2 risk variants carry potential to increase ER stress in podocytes.

### Suppression of ER stress attenuates APOL1 risk variants-induced podocyte injury

**Figure 7:**
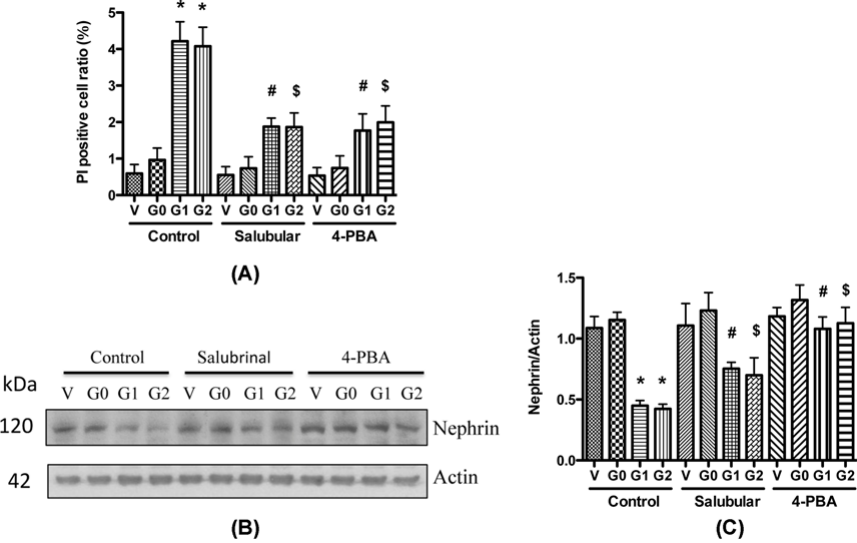
ER stress inhibitors attenuate APOL1 risk variants caused podocyte injury (**A**) Differentiated HPs (APOL1-Vec/HPs, G0/HPs, G1/HPs, and G2/HPs) were treated with Salubrinal (2.5 μM) or 4-PBA (1 mM) for 48 h, and cell lysates were collected for PI staining. Necrotic cells were counted, and the cell ratio was calculated. *, ^#^, and ^$^, *P*<0.05 in comparison with APOL1-G0, APOL1-G1, and APOL1-G2 in control group, respectively. (**B**,**C**) Differentiated HPs (APOL1-Vec/HPs, G0/HPs, G1/HPs, and G2/HPs) were treated with Salubrinal (2.5 μM) or 4-PBA (1 mM) for 48 h, and cell lysates were collected for Western blotting to detect nephrin protein. Representative gels are shown in (**B**). The protein bands were scanned and the acquired images were analyzed using the public domain NIH image program for data quantitation. Expression of nephrin (**C**) were normalized to β-actin. Data are presented as fold of control expression. *, ^#^, and ^$^, *P*<0.05 in comparison with APOL1-G0, APOL1-G1, and APOL1-G2 in control group, respectively.

To assess a possible causal relationship between the increased ER stress and podocyte injury, we examined the effect of ER stress on podocyte necrosis by adding ER stress inhibitor PBA and eIF-2α inhibitor salubrinal to the medium. We found that both inhibitors significantly decreased the PI positive cell numbers in G1 and G2 expressing podocytes (Figure 7A). These results demonstrate that suppressing ER stress could attenuate APOL1 risk variants-induced necrosis in podocytes.

We also examined the effect of ER stress inhibitors on the expression of nephrin in APOL1G0 and variants expressing podocytes with 4-PBA and salubrinal. After 48 h, the cell lysates were collected for Western blotting. Both 4-PBA and salubrinal increased the nephrin protein expression in APOL1 G1/G2-expressing podocytes at 48 h post treatment (Figure 7B,C). We also evaluated the effect of ER stress inhibitors on the alteration of mRNA levels; real time-PCR results showed that the mRNA level did not change (data not shown). These results indicate that blocking of ER stress carries potential of attenuation of APOL1 G1/G2-mediated protein translation but not the transcription.

In the present study, we found that APOL1 risk variants significantly increased the expression of GRP78, the molecular chaperone in ER stress, indicating that the ER stress was increased by APOL1 G1 and G2, but not by G0. Moreover, the cells expressing the APOL1 G1 or G2 exhibited a significant increase in the phosphorylation of eIF-2α. Consistently, only the cells overexpressing APOL1 G1 and G2 variants inhibited the translation of nephrin and podocin. ER stress and eIF-2α inhibitors not only partially attenuated a decrease in nephrin and podocin expression but also provided protection against necrosis.

In summary, APOL1 risk variants contribute to podocyte injury through enhanced ER stress. Our study provides insight into new mechanisms involved in APOL1 risk variants-induced podocyte damage, and highlights some new therapeutic targets for APOL1-associated nephropathy.

## Abbreviations

APOL1: apolipoprotein L1
BiP: binding Ig protein
CKD: chronic kidney disease
ER: endoplasmic reticulum
FSCS: focal segmental glomerulosclerosis
GRP78: glucose-regulated protein 78
HIVAN: HIV-associated nephropathy
HP: human podocyte
ITS: insulin, transferrin, and selenium
PERK: protein kinase-like ER kinase
PI: propidium iodide
SD: slit diaphragm
UPR: unfolded protein response
4-PBA: 4-phenylbutyrate
